# Pathogenetic Potential Relating to Metabolic Activity in a Mouse Model of Infection with the Chikungunya Virus East/Central/South African Genotype

**DOI:** 10.3390/v12020169

**Published:** 2020-02-03

**Authors:** Mya Myat Ngwe Tun, Rohitha Muthugala, Aung Kyaw Kyaw, Satoshi Shimada, Kouichi Morita, Daisuke Hayasaka

**Affiliations:** 1 Department of Virology, Institute of Tropical Medicine, Nagasaki University, Nagasaki 852-8523, Japan; myamyat@tm.nagasaki-u.ac.jp (M.M.N.T.); rohithavm@yahoo.com (R.M.); kanomkrok77@gmail.com (S.S.); moritak@nagasaki-u.ac.jp (K.M.); 2 Leading Graduate School Program, Nagasaki University, Nagasaki 852-8523, Japan; 3 Laboratory of Veterinary Microbiology, Joint Faculty of Veterinary Medicine, Yamaguchi University, Yamaguchi 753-8515, Japan

**Keywords:** CHIKV, ECSA, IFN-γ, mouse model, viral pathogenesis

## Abstract

Epidemics of the Chikungunya virus (CHIKV) from 2004 onwards were caused by the East/Central/South African (ECSA) genotype. However, the pathogenesis of the genotype infection has not been fully explained. In this study, we examined the pathogenic potential of CHIKV ECSA genotype M-30 (M-30) by comparing it with that of African genotype S-27 (S-27) in mice. Following low titer infections in type-I IFN receptor KO (A129) mice, we found that the M-30 infection caused high and acute fatality compared with the S-27 infection. M-30-infected A129 mice showed higher viral loads in their central nervous systems and peripheral organs, and increased levels of IFN-γ responses in their brains. Interestingly, M-30-infected mice did not show the hypophagia and reductions in weight which were observed in S-27-infected mice. Our observations provide a novel explanation of the pathogenic mechanisms attributed to virus proliferation, anti-type-II IFN response and metabolic activity in the CHIKV ECSA virus in mice.

## 1. Introduction

Chikungunya virus (CHIKV) belongs to the genus Alphavirus (family Togaviridae) and is transmitted to humans via the bite of infected mosquitoes such as Aedes aegypti and Aedes albopictus [1]. The CHIKV genome is a single-stranded, positive-sense RNA virus with a length of approximately 12 kb [2]. The genome is composed of nonstructural proteins (5′-nsP1-nsP2-nsP3-nsP4) and structural proteins (C-E3-E2-6K-E1-3′) [3]. CHIKV strains are clustered into three distinct genotypes: West African, East/Central/South African (ECSA) and Asian [4].

Historically, it has been recognized that CHIKV infection causes similar clinical symptoms to dengue fever: fever, rash and arthritis. During 2005 and 2006, Chikungunya fever emerged as a serious epidemic in India [5]. This epidemic is believed to have originated in Kenya in 2004 [6]. The massive epidemic spread to several Asian countries, Europe and the United States between 2005 and 2010 [4]. It has been found that the outbreaks from 2004 onwards have been caused by the ECSA genotype of CHIKV. These outbreaks displayed unusual clinical severity including neurological disorders, mother-to-child transmission and deaths [7,8]. However, the pathogenicity of the ECSA genotype causing severe symptoms such as neurological complications has not been fully explained.

Langsjoen et al., 2018 reported that Asian/American CHIKV strains are less virulent than those in the Asian, ECSA and West African genotypes and that despite differences in virulence, Indian Ocean Lineage-based vaccine strains offer a robust cross-protection against strains from other lineages [9]. In 2011, Partidos et al. reported that the importance of IFNs in controlling the CHIK 181/25 vaccine and demonstrated the ability of this vaccine to elicit neutralizing antibody responses that confer short-and long-term protection against wild type CHIKV-La Reunion challenge [10]. Our study aimed to report the pathogenetic potential relating to metabolic activity and anti-type II IFN response in a mouse model by using two different strains (S-27 and M-30) of the same genotype (ECSA).

Interestingly, our preliminary experiments show that in human cell lines, the ECSA genotype M-30 strain (M-30), which was isolated from a clinical patient, replicated at a significantly higher rate than the African genotype strain S-27 (S-27) (unpublished data). From these observations, we suggest that comparisons of the pathogenic properties of the M-30 and S-27 infections may provide valuable clues to elucidate the specific pathogenicity of the ECSA genotype.

To examine the mechanism of the pathogenicity of alphavirus infections such as CHIKV, Sindbis virus, Semliki Forest virus and Ross River virus, a laboratory mouse model has commonly been employed [11,12,13,14,15,16]. In the present study, we examined and compared the pathogenic potential of the CHIKV ECSA genotype of the M-30 infection by comparing it with that of the prototype S-27 infection using an in vivo mouse model.

## 2. Materials and Methods

### 2.1. Virus and Cells

CHIKV M-30 (KF 590566.1) was isolated from the acute serum (4 days from illness) of a 6 year-old female child examined in our previous study who had dengue fever in Myanmar in [17], and S-27 (AF 369024.2) was isolated from a febrile patient in Tanzania in 1952 [2]. These viruses were propagated in *Aedes albopticus* mosquito cell line C6/36 and the culture fluids were used after a few passages. C6/36 cells were maintained in minimum essential medium supplemented with 2% fetal calf serum. The cells were grown at 28 °C. Stock viruses of the CHIKV M-30 and S-27 strains were prepared in C6/36 cells. All experiments using live CHIKV were performed in a biosafety level 3 laboratory at the Institute of Tropical Medicine, Nagasaki University, according to the standard BSL3 guidelines.

### 2.2. Mice

The A129 and AG129 mice were purchased from B & K Universal Limited. These mice were mated in the Nagasaki University facility. Ten mice were subcutaneously inoculated with 10^2^ or 10^5^ plaque forming units (PFU) of S-27 and M-30. The mice were weighed daily and observed for clinical signs for 21 days. The experimental protocols were approved by the Animal Care and Use Committee of Nagasaki University (approval number: 141201115-5, approval date: 9 March 2015). 

### 2.3. Virus Titration

The A129 and AG129 mice subcutaneously inoculated with 10^5^ and 10^2^ pfu of M-30 and S-27 were euthanized and sacrificed. The blood was collected and after perfusion with cold phosphate-buffered saline, the thymus, lungs, heart, liver, spleen, stomach, small intestine, large intestine, kidneys, muscle, brain, and spinal cord were also collected. The brains were divided into two parts: the brain cortex (brain-1) and other parts (brain-2). Both parts were kept at –80°C until further use. Each tissue was homogenized and virus titers were determined by plaque-forming assays in cells and were expressed as pfu/g tissue [18].

### 2.4. Quantification of Inflammatory Cytokines Using Real-Time Polymerase Chain Reaction

As discussed above, following CHIKV infection, the mice were sacrificed and their spleens and brains were collected after perfusion. Those tissues were immediately submerged in RNAlater (Life Technologies, Carlsbad, CA, USA). Total RNA was extracted using an RNeasy Lipid Tissue Mini Kit (Qiagen, Valencia, CA, USA). Transcribed mRNA levels of IFN-γ, IL-2, IL-4, IL-6, IL-10 and TNF-α were examined using a SYBR real-time polymerase chain reaction (PCR), as demonstrated previously [19,20]. The absolute copy numbers of unknown samples were calculated by comparing the threshold cycle with the corresponding standard curve [21].

### 2.5. Measurement of Cytokines Levels Determined by a Milliplex Map Kit

Serum samples were collected from mice infected with CHIKV (10^2^ FFU) and from mock-infected mice at 5 days post-infection (pi). The cytokines, IFN-γ, IL-2, IL-4, IL-6, IL-10, IL-12P40, MCP-1 and TNF-α levels were measured using a Magnetic bead Milliplex Kit (Millipore, Billerica, MA, USA) according to the manufacturer’s instructions. The plate was read on Luminex-200^TM^ with xPONENT software (Luminex corporation, Austin, Texas, USA).

### 2.6. Quantification of Inflammatory Cytokines Using Real-Time Polymerase Chain Reaction

The Kruskal–Wallis Mann–Whitney U-tests were used to assess the significant differences in viral loads, mRNA and protein levels of cytokines. A one way ANOVA test and a Student’s *t*-test were used to assess any significant differences in food and water intakes.

## 3. Results

### 3.1. M-30 Infection Caused More Severe Disease than S-27 Infection in A129 Mice

We first attempted to infect immunocompetent mice such as C57BL/6 and BALB/c mice with CHIKV. However, these mice did not exhibit any apparent clinical signs and none of them died. Therefore, we then attempted to infect type-I and/or type-II IFN function deficient mice which are sensitive to viral infections.

**Figure 1:**
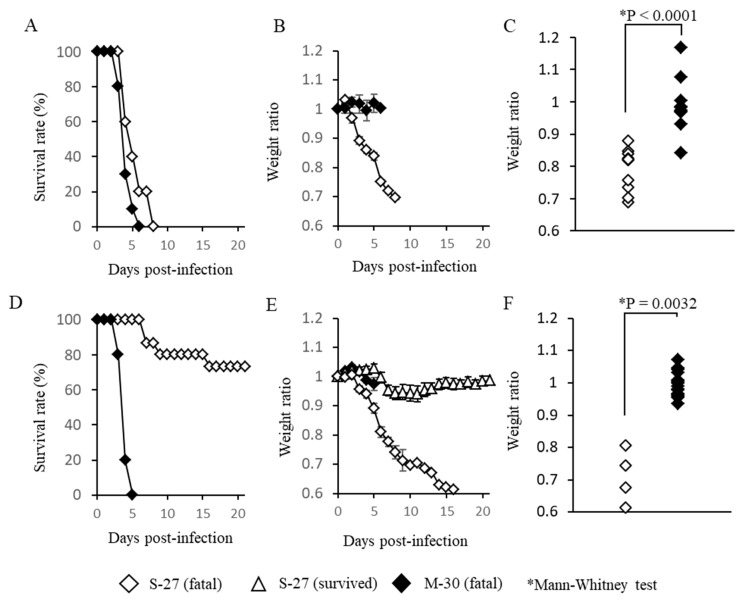
Survival rates (**A**,**D**), average ratios of weight change (**B**,**E**) and individual weights when the mice died (**C**,**F**) of A129 mice subcutaneously infected with high titer 10^5^ PFU (**A**–**C**) and low titer 10^2^ PFU (**D**–**F**) of M-30 and S-27 CHIKV strains. The error bars represent the standard deviations. The asterisks show significant differences by the Mann–Whitney U-test, *P* < 0.05.

A129 mice that lacked the functions of a type-I IFN receptor were infected with high titer (10^5^ pfu) or low titer (10^2^ pfu) of M-30 and S-27 and their clinical courses were observed (Figure 1). Following infection with high titer infections of M-30 and S-27, all mice died (Figure 1A). Their survival times were 4.2 ± 0.57 and 5.4 ± 0.98 days, respectively. The survival times of M-30-infected mice were approximately one day shorter than those of S-27-infected mice, although they were not statistically different. Interestingly, the weight of M-30-infected mice was not reduced by the time the mice died, whereas S-27-infected mice exhibited weight reductions (Figure 1B). Consequently, individual weights when the mice died were significantly different (*P* < 0.0001) between M-30 and S-27 infections (Figure 1C).

Following low titer infections, fatalities of M-30- and S-27-infected mice were 100% and 26.7%, respectively (Figure 1D). The average survival times of fatal mice were 4.0 ± 0.33 and 9.8 ± 4.2 days, respectively. Of note, the weights of M-30-infected mice were not reduced during disease progression, whereas fatal cases of S-27-infected mice exhibited higher weight loss, similarly to those with high titer infections (Figure 1E). The surviving mice infected with the S-27 virus showed slight weight reductions and then recovered (Figure 1E). Consequently, the individual weights of fatal mice when they died were significantly different (*p* = 0.0032) between the M-30 and S-27 infections (Figure 1F).

These observations show that in A129 mice: (i) infections with high titers of both the M-30 and S-27 viruses caused fatal infections, indicating that high titer infections determine a lethal outcome; but (ii) under low titer infections, the M-30 infection caused high and acute fatality compared with the S-27 infection; and (iii) clinical signs relating to weight changes were clearly different between the M-30 and S-27 infections. These results suggest that the ECSA M-30 infection causes a more severe disease course than the classical prototype S-27 infection.

### 3.2. The M-30 and S-27 Infections Were Fatal in AG129 Mice

**Figure 2:**
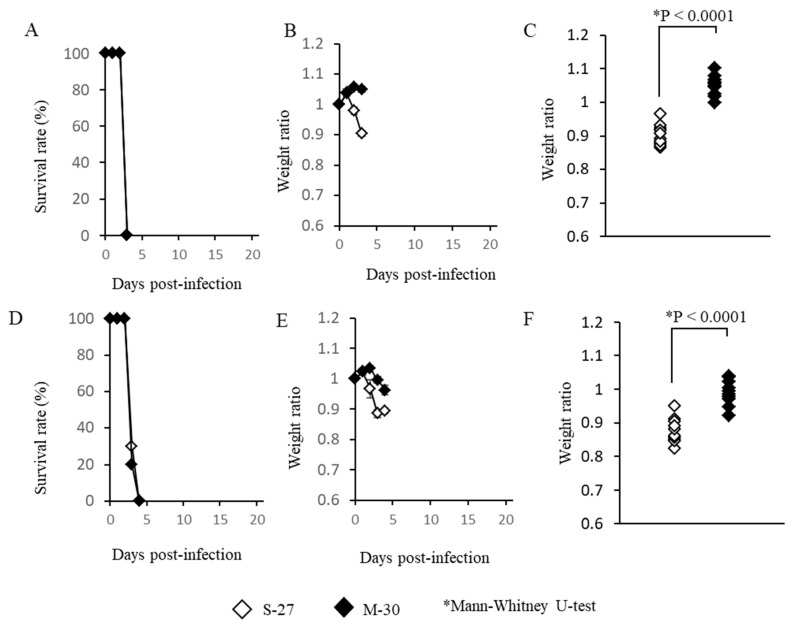
Survival rates (**A**,**D**), average ratios of weight change (**B**,**E**) and individual weights when the mice died (**C**,**F**) of AG129 mice following subcutaneous infection with high titer 10^5^ PFU (**A**–**C**) and low titer 10^2^ PFU (**D**–**F**) of M-30 and S-27 CHIKV strains. The error bars represent the standard deviations. The asterisks show significant differences by the Mann–Whitney U-test, *P* < 0.05.

AG129 mice, which lack functioning type-I and -II IFN receptors, were also infected with high titer (10^5^ pfu) or low titer (10^2^ pfu) of the M-30 and S-27 viruses and their clinical courses were observed (Figure 2). High titer infections with both viruses induced 100% fatality at 3 days pi (Figure 2A). The M-30-infected mice showed lower weight reductions than the S-27-infected mice (Figure 2B), and the weights of fatal mice were significantly different (*p* < 0.0001) between the S-27 and M-30 infections (Figure 2C).

Low titer infections also caused 100% fatality in both the M-30- and S-27-infected mice (Figure 2D) and their survival times were 3.2 ± 0.26 and 3.3 ± 0.30 days, respectively. The M-30-infected mice showed lower weight reductions than the S-27-infected AG129 mice (Figure 2E), and the weights of lethal mice were significantly different (*p* < 0.0001) between the M-30 and S-27 infections (Figure 2F), similarly to the high titer infections.

These observations show that the M-30 and S-27 infections caused 100% fatality in A129 and AG129 mice, although clinical signs relating to weight change were different. Based on these results and the results relating to the A129 mice, it is suggested that type-II IFN responses exert potential protective effects against the fatal S-27 virus but not M-30 infections. In other words, the M-30 infection induces resistant responses to type-II IFN effects compared with the S-27 infection.

### 3.3. The M-30-Infected Mice Did Not Develop Hypophagia

**Figure 3:**
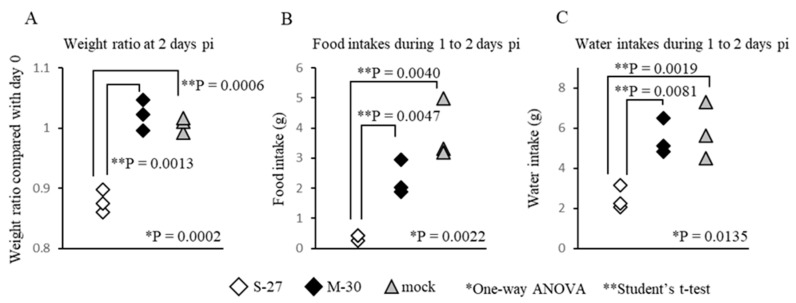
(**A**) The ratio of weight change, (**B**) food intakes, (**C**) water intakes for individual AG129 mice following subcutaneous infection with high titer 10^5^ PFU M-30 and S-27 CHIKV strains. The asterisks show significant differences by one-way ANOVA (*) and by the Student’s t-test (**), *P* < 0.05.

To ascertain the cause of the different weight changes between M-30- and S-27-infected mice, food and water intakes were compared in AG129 mice infected with 10^5^ PFU (Figure 3). Under these infectious conditions, the mice infected with both viruses died at 3 days pi, but showed clearly different (*p* = 0.0013) weight ratios (Figure 3A) at 2 days pi. Interestingly, the food and water intakes of M-30-infected mice were similar to those of the mock-infected mice during 1 to 2 days pi, whereas those of S-27-infected mice were significantly (*p* = 0.0047 in food intake, *p* = 0.0081 in water intake) reduced (Figure 3B,C).

Therefore, the body-weight changes were clearly related to food and water intake, indicating that the M-30-infected mice had appetites, whereas the S-27-infected mice did not. These observations suggest that M-30 infection does not cause the weight reduction typically observed in pathogenic viral infections.

### 3.4. Viral Propagation Was Related to Severe Disease Caused by the M-30 Infection in A129 Mice

**Figure 4:**
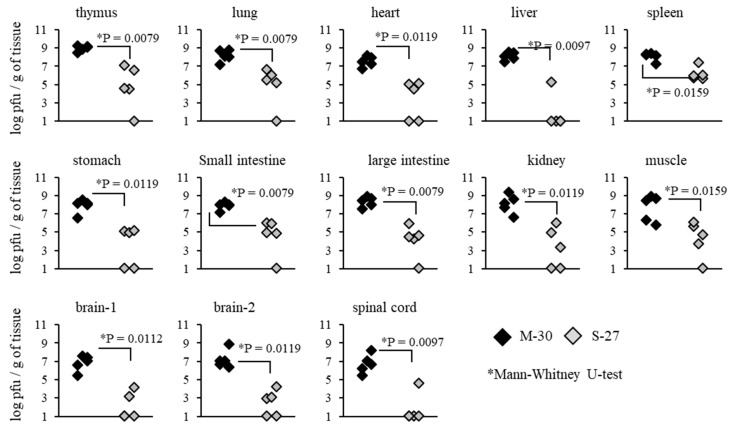
Viral loads in tissues of A129 mice at 3 days after subcutaneous infection with 10^2^ PFU of the M-30 and S-27 CHIKV strains. The asterisks show significant differences by the Mann–Whitney U-test, *P* < 0.05.

To investigate whether severe diseases caused by the M-30 infection were related to viral propagation in mice, viral loads in the organs and tissues of A129 mice were determined following infection with 10^2^ PFU (Figure 4). Compared with the S-27-infected mice, M-30-infected mice showed significantly higher viral loads in the thymus (*p* = 0.0079), lungs (*p* = 0.0079), heart (*p* = 0.0119), liver (*p* = 0.0097), spleen (*p* = 0.0159), stomach (*p* = 0.0119), small intestine (*p* = 0.0079), large intestine (*p* = 0.0079), kidneys (*p* = 0.0119), muscle (*p* = 0.0159), brain 1 (*p* = 0.0112), brain 2 (*p* = 0.0119) and spinal cord (*p* = 0.0097) at 3 days pi (Figure 4).

**Figure 5:**
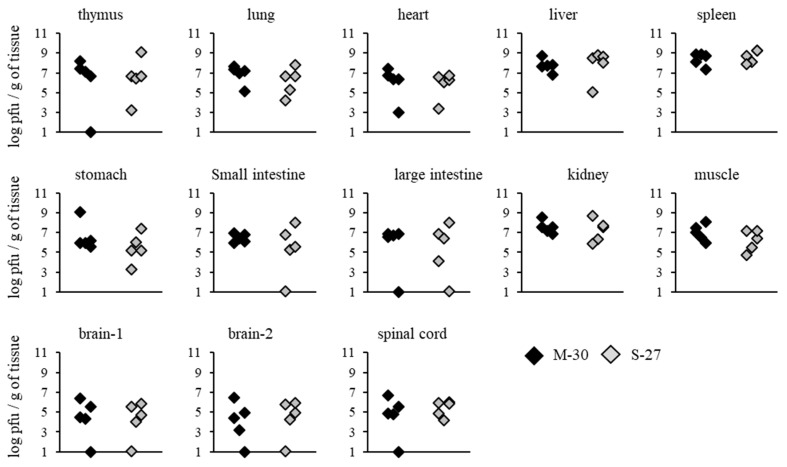
Viral loads in tissues of AG129 mice at 2 days after subcutaneous infection with 10^2^ PFU of the M-30 and S-27 CHIKV strains.

On the other hand, viral loads in AG129 mice were not significantly different between the M-30 and S-27 infections at 2 days pi (Figure 5). These results suggest that viral propagations in the peripheral and central nervous system (CNS) tissues are reflected in the higher clinical severity of M-30-infected mice and that the type-II IFN response exerts more potential effect against the viral proliferation of the S-27 infection than the M-30 infection.

### 3.5. Distinct Inflammatory Responses in M-30-Infected Mice

**Figure 6:**
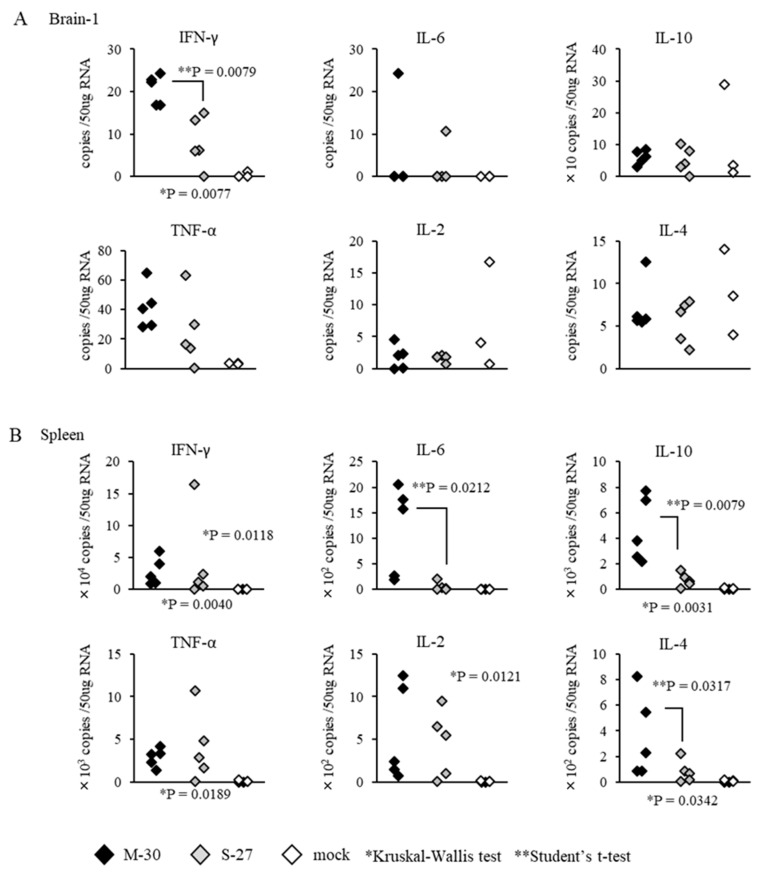
mRNA levels of IFN-γ, IL-6, IL-10, TNF-α, IL-2, and IL-4 quantified using real-time PCR in the brains (**A**) and spleens (**B**) of A129 mice at 3 days after subcutaneous infection with 10^2^ PFU of M-30 and S-27 CHIKV strains. The asterisks show significant differences by the Kruskal–Wallis test (*) and by the Mann–Whitney U-test (**), *P* < 0.05.

To examine whether distinct inflammatory responses were induced in the M-30 and S-27 infections, the mRNA levels of major cytokines in the CNS (brain cortex) and peripheral (spleen) tissues were determined in both A129 mice infected with 10^2^ PFU of both viruses at 3 days pi and mock-infected mice (Figure 6). The levels of IFN-γ were significantly higher (*p* = 0.0079) in the brains of M-30-infected mice than in those of the S-27-infected mice, and other cytokine levels in the brains were not significantly different between the M-30- and S-27-infected mice (Figure 6A). Levels of IFN- γ (*p* = 0.0118), IL-6 (*p* = 0.0212), IL-10 (*p* = 0.0079), IL-2 (*p* = 0.0121) and IL-4 (*p* = 0.0317) were significantly higher in the spleens of M-30-infected mice than in those of the S-27-infected mice, andTNF-α levels were not significantly different between the M-30 and S-27 infections (Figure 6B).

**Figure 7:**
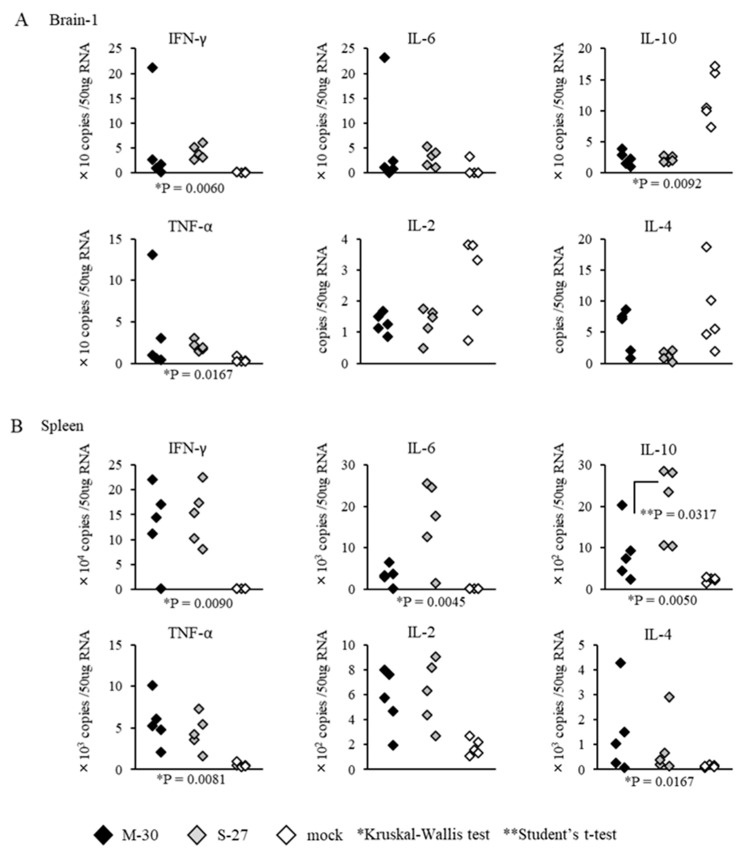
mRNA levels of IFN-γ, IL-6, IL-10, TNF-α, IL-2, and IL-4 quantified using real-time PCR in the brains (**A**) and spleens (**B**) of AG129 mice at 2 days after subcutaneous infection with 10^2^ PFU of M-30 and S-27 CHIKV strains. The asterisks show significant differences by the Kruskal–Wallis test (*) and by the Mann–Whitney U-test (**), *P* < 0.05.

The inflammatory responses of AG129 mice were also observed following infections with 10^2^ PFU of the M-30 and S-27 viruses (Figure 7). Cytokine levels in the brains and spleens (except IL-10 (*p* = 0.0317) in the spleens) were not significantly different between M-30- and S-27-infected mice (Figure 7A,B).

**Figure 8:**
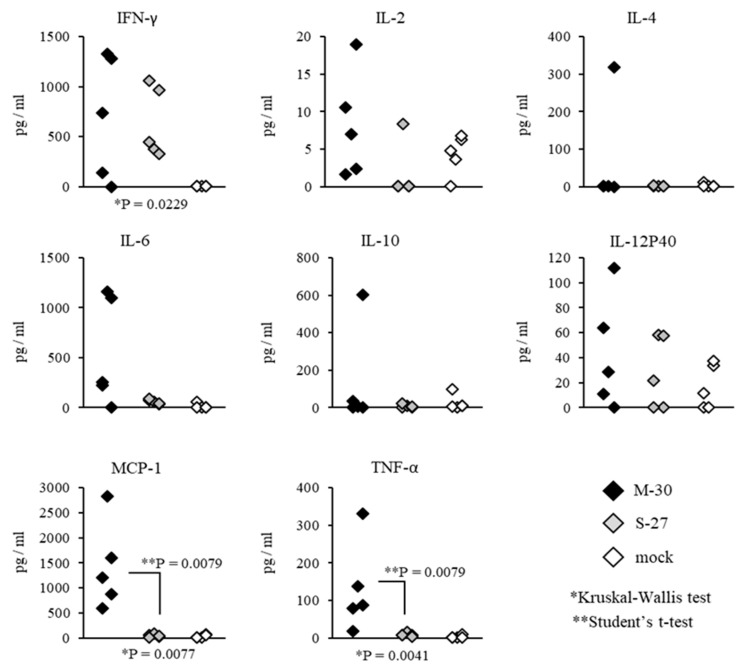
Serum cytokine levels of IFN-γ, IL-2, IL-4, IL-6, IL-10, IL-12P40, MCP-1 and TNF-α were determined by multiplex magnetic bead assay in A129 mice at 3 days after subcutaneous infection with 10^2^ PFU of M-30 and S-27 CHIKV strains. The asterisks show significant differences by the Kruskal–Wallis test (*) and by the Mann–Whitney U-test (**), *P* < 0.05.

**Figure 9:**
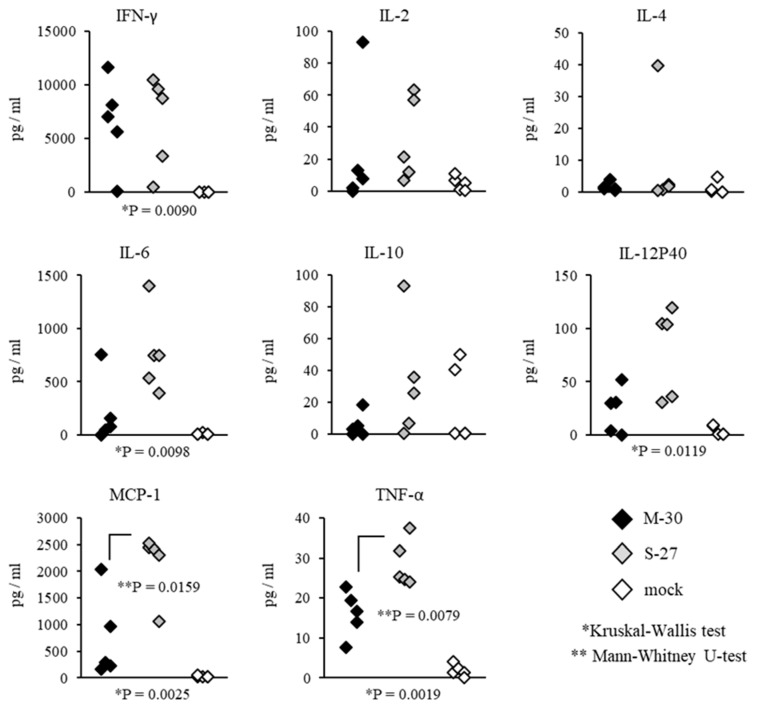
Serum cytokine levels of IFN-γ, IL-2, IL-4, IL-6, IL-10, IL-12P40, MCP-1 and TNF-α were determined by multiplex magnetic bead assay in AG129 mice at 2 days after subcutaneous infection with 10^2^ PFU of M-30 and S-27 CHIKV strains. Asterisks show significant differences by Kruskal-Wallis test (*) and by Mann–Whitney U-test (**), *P* < 0.05.

Systemic inflammatory responses of CHIKV-infected mice were also compared by determining the representative cytokine levels in the serum (Figure 8 and Figure 9). In A129 mice, the levels of MCP-1 (*p* = 0.0079) and TNF-α (*p* = 0.0079) were significantly increased in M-30-infected mice compared with S-27-infected and mock-infected mice (Figure 8).

On the other hand, in AG129 mice, the levels of MCP-1 (*p* = 0.0159) and TNF-α (*p* = 0.0079) were significantly increased in S-27-infected mice compared with M-30-infected and mock-infected mice (Figure 9). The levels of IFN-γ, IL-2, IL-4, IL-6, IL-10 and IL-12P40 were not significantly different between M-30- and S-27-infected mice (Figure 8 and Figure 9).

These results suggest that distinct inflammatory responses may relate to severe diseases causing 100% fatality due to the M-30 infection compared with the S-27 infection, although it is not clear whether these responses were the causes or results of the severe diseases.

## 4. Discussion

In this study, we demonstrated that the CHIKV ECSA genotype M-30 has a higher pathogenetic potential than the African genotype S-27 in an in vivo mouse model. We suggest that this higher pathogenicity is associated with type-II IFN responses, as observed in the different fatality levels of A129 and AG129 mice.

Interestingly, M-30-infected mice did not experience hypophagia or weight reductions. As far as we know, this phenomenon is unique among the clinical signs that have been observed in viral infections because pathogenic virus inoculations usually induce weight loss in the experimental mice models during disease development. From our experience, we also consider that weight reduction is an important key factor in the pathogenicity of viral infections [17,18,21,22]. Our findings raise the possibility that the M-30 infection may induce atypical metabolic activity. Therefore, our established mouse model of CHIKV infections may provide a novel concept of the pathogenic mechanism related to metabolic activity and viral infections.

The sequence identities of nucleotides and amino acids between the M-30 and S-27 infections were 98.2% identical. Among the 60 amino acid differences, 26 structural proteins (three positions in C, 2 in E3, 15 in E2, 2 in 6K and four in E1) and 34 non-structural proteins (9 in NSP1, 6 in NSP2, 13 in NSP3 and 6 in NSP4), changes were identified. Previous studies have shown that the A226V mutation in the E1 protein identified in ECSA genotype strains increased its infectivity in human and mouse cell lines and contributed to more efficient dissemination and transmission by *Aedes albopictus* [23,24,25]. Only the M-30, and not the S-27 infection, has the specific A226V mutation in the genome. Although it is currently unknown whether a single or multiple viral genome mutation(s) contribute to the specific pathogenicity of ECSA CHIKV, determination of the viral genes responsible for higher pathogenicity in the M-30 genome in our mice model will provide potential clues to the viral factors of ECSA pathogenicity.

The interferon response is the first line of defense against viral infections, suppressing the replication of viruses and their spread [26]. Similarly to other alphaviruses, CHIKV replication caused a dramatic shutoff of host gene expression, resulting in the suppression of innate immunity [27]. Our results show that interferon responses played a critical role in preventing lethal infections of CHIKV because IFN responses of deficient A129 and AG129 mice exhibited lethal infections, but immunocompetent mice did not. Further, our results show that the M-30 infection was more resistant to the protective effects of type-II IFN responses than the S-27 infection because low titer infections of M-30, but not S-27, induced 100% lethality in A129 mice. Thus, we suggest that determining the specific viral gene product(s) of M-30 relating to type-II IFN responses would be useful in identifying the viral factors against type-II IFN responses in CHIKV infections in vivo. Further studies, such as in vitro analyses using some cultured cell lines, may provide further clues to interpreting the mechanism of different pathogenicities relating to the IFN responses observed in M-30 and S-27 infections in mice. 

High titers of both M-30 and S-27 infections in A129 mice proved fatal, whilst viral loads were considered to be sound predictors of a lethal outcome. In A129 mice infected with low titers, viral loads in the peripheral and CNS tissues were significantly increased in M-30-infected mice compared with S-27-infected mice. In addition, following infections with both M-30 and S-27, viral loads in AG129 mice were substantially higher than those in A129 mice. These observations indicate that viral loads are closely correlated with severe pathogenicity and fatality in mice. However, it is unclear whether viral proliferation is a cause or a result of severe diseases caused by fatal infection. In A129 mice, different inflammatory responses were observed following infections with low titers of M-30 compared with S-27. These immune responses were detected via mRNA levels of IFN-γ in the brain and of IL-6 and IL-10 in the spleen and protein levels of IL-6, MCP-1 and TNF-α in the plasma. Thus, we suggest that those distinct inflammatory responses may be correlated with severe pathogenicity and fatality in mice. However, currently, it is unclear whether these responses have a protective or promotive effect on viral proliferation or whether they are a cause or a result of severe diseases caused by fatal infections. Further analysis is required to answer these questions which may provide useful information to help elucidate the pathogenic mechanism of CHIKV infections.

In conclusion, our observations raise the concern that the pathogenesis of ECSA may be explained by a complex mechanism. Thus, we suggest that the elucidation of the precise mechanism of the pathogenesis of ECSA requires a multistep approach, including the identification of the host factors such as metabolic activity and IFN responses and the determination of viral factors based on the viral genome specific to M-30 using the infectious cDNA technique.
